# The role of universal health coverage and global health security nexus and interplay on SARS-CoV-2 infection and case-fatality rates in Africa : a structural equation modeling approach

**DOI:** 10.1186/s12992-023-00949-2

**Published:** 2023-07-06

**Authors:** Sibhatu Biadgilign, Alemayehu Hailu, Bereket Gebremichael, Mekitew Letebo, Etsub Berhanesilassie, Arega Shumetie

**Affiliations:** 1 Independent Public Health Analyst and Research Consultant, P.O.BOX 24414, Addis Ababa, Ethiopia; 2 Department of Global Public Health and Primary Care Medicine, Bergen Center for Ethics and Priority Setting, The University of Bergen, Bergen, Norway; 3 Harvard T.H. Chan School of Public Health, Harvard University, Boston, United States of America; 4 College of Health Science, Addis Ababa University, Addis Ababa, Ethiopia; 5 Ethiopian Economics Association, Addis Ababa, Ethiopia

**Keywords:** UHC, GHS, SARS-CoV-2, Africa, Infection rate, Case fatality rate, Mortality rate

## Abstract

**Background:**

The Coronavirus Disease (COVID-19) caused by SARS-CoV-2 infections remains a significant health challenge worldwide. There is paucity of evidence on the influence of the universal health coverage (UHC) and global health security (GHS) nexus on SARS-CoV-2 infection risk and outcomes. This study aimed to investigate the effects of UHC and GHS nexus and interplay on SARS-CoV-2 infection rate and case-fatality rates (CFR) in Africa.

**Methods:**

The study employed descriptive methods to analyze the data drawn from multiple sources as well used structural equation modeling (SEM) with maximum likelihood estimation to model and assess the relationships between independent and dependent variables by performing path analysis.

**Results:**

In Africa, 100% and 18% of the effects of GHS on SARS-CoV-2 infection and RT-PCR CFR, respectively were direct. Increased SARS-CoV-2 CFR was associated with median age of the national population (β = -0.1244, [95% CI: -0.24, -0.01], *P* = 0.031 ); COVID-19 infection rate (β = -0.370, [95% CI: -0.66, -0.08], *P* = 0.012 ); and prevalence of obesity among adults aged 18 + years (β = 0.128, [95% CI: 0.06,0.20], *P* = 0.0001) were statistically significant. SARS-CoV-2 infection rates were strongly linked to median age of the national population (β = 0.118, [95% CI: 0.02,0.22 ], *P* = 0.024); population density per square kilometer, (β = -0.003, [95% CI: -0.0058, -0.00059], *P* = 0.016 ) and UHC for service coverage index (β = 0.089, [95% CI: 0.04,0.14, *P* = 0.001 ) in which their relationship was statistically significant.

**Conclusions:**

The study shade a light that UHC for service coverage, and median age of the national population, population density have significant effect on COVID-19 infection rate while COVID-19 infection rate, median age of the national population and prevalence of obesity among adults aged 18 + years were associated with COVID-19 case-fatality rate. Both, UHC and GHS do not emerge to protect against COVID-19-related case fatality rate.

**Supplementary Information:**

The online version contains supplementary material available at 10.1186/s12992-023-00949-2.

## Introduction

Coronavirus disease 2019 (COVID-19) was declared as a global pandemic by the World Health Organization (WHO) on March 11, 2020. It is affecting all continent across the globe [1–3] and is described as the greatest global health threat [4]. As of May 4, 2022 globally, over 3.8 million cases and over 15 000 deaths were reported during the week of 25 April through 1 May 2022, in which a decreases 17% and 3% respectively. The cumulative number of confirmed cases reported globally is now over 500 million and the cumulative number of deaths is more than 6 million as of 1 May 2022 [5]. COVID-19 potentially being a leading cause of mortality in 2020 and 2021 [6]. Low middle income countries (LMIC) may experience far worse mortalities considering the existence of a weak health care system and several underlying population level morbidities. As a result, it becomes evidenced to understand the ecological correlation between critical underlying population level morbidities and COVID-19 case fatality rates (CFR) [7]. The actual number of infections in many countries remains underestimated and under-reported due to the asymptomatic nature of the infection and inadequate testing and surveillance system [8].

The COVID-19 pandemic has placed enormous strain on countries around the world, exposing long-standing gaps in public health and exacerbating chronic inequities. By assessing the ability of health systems to manage and analyze the spread of COVID-19 from the perspective of two key approaches to global health policy—global health security (GHS) and universal health coverage (UHC)—important lessons can be drawn for how to align varied priorities and objectives in strengthening health systems and fostering resilience moving forward [9, 10]. The GHS index is the first comprehensive assessment of countries’ preparedness for outbreaks response through evaluating the health security and related capabilities of 195 countries that are Parties to the International Health Regulations (2005) [11]. UHC depends on access to comprehensive, appropriate, timely, and quality health services, without financial burden. Although UHC enables primary health care(PHC) systems and improves the accessibility of health services, in practice there is a tendency for UHC interventions to neglect infectious disease threats and inadequately manage the core capacities of public health while focusing more on health insurance and individual health services [10, 12–14]. Meanwhile, access to essential services represents the lowest capacity in most countries of the WHO African region, specifically due to poor physical access to services [15]. The resilience of a health system, as an example from Ebola-affected countries, need efforts to not only restore how the system functioned before the crisis but to transform and fundamentally improve the health system [16, 17]. In addition to this, the wider lessons from the Ebola epidemic emphasize the urgency to reach consensus on transformative initiatives with the potential to mitigate risks of another emergency similar to that of Ebola [17, 18]. A system with strategies for social protection, cost-effective PHC, inclusive leadership, and adequate public financing can guarantee quality services for all, especially in fragile contexts where poverty, overcrowded housing, and inadequate resources make communities most susceptible [9, 19]. The COVID-19 pandemic highlights how fragmented and underfunded health systems are globally [9], with more disruption in the health system being seen in Africa, where the health system is already characterized by weak, folded in crisis with nations that are generally impacted by armed conflict [20–23]. As the pandemic continues, healthcare systems may be overwhelmed in Africa demonstrated by skilled healthcare workers are in short supply and 1–5% of the intensive care unit beds per capita [23]. There is a paucity of evidence on the nexus and interplay of universal health coverage and global health security on the dynamics of the COVID-19 infection and case-fatality rates. Within this stand in mind, the aim of this study was to investigate the role of universal health coverage and global health security nexus and interplay on COVID-19 infection and case-fatality rates in Africa.

## Methods

### Study design and population

In this ecological study, we investigate the role of UHC and GHS nexus and interplay on COVID-19 infection and case-fatality rates in Africa. The list of African countries included in this study is attached in the supplementary file (Additional file 1). The study followed the Strengthening the Reporting of Observational Studies in Epidemiology (STROBE) reporting guideline.

### Data collection and extraction

Data for this paper was drawn from multiple sources, wherein epidemiological data on COVID-19 cases, deaths, and recoveries were collected from the international databases on the Worldometer website as of June 26, 2021 [24]. Only laboratory diagnosis and confirmed cases of COVID-19 infection using real-time reverse‐transcriptase‐polymerase chain reaction [RT‐PCR]) were used. COVID-19 data have been reported on a daily basis for all countries across the globe, in which this particular study focus on 54 African countries wherein the data are available and included in the analysis. Country-level data on the preparedness to prevent, detect, respond, health, norms and risk to infectious disease threats using the Global Health Security (GHS) index (https://www.ghsindex.org) [25]. Data for each country-level analysis were obtained from the Our World in Data (https://ourworldindata.org/) [26]; World Health Organization (WHO) Global Health Observatory Repository (https://www.who.int/data/gho) [27] and the World Bank’s World Development Indicators (https://databank.worldbank.org/source/world-development-indicators) [28].

### Variables used in the study

#### Dependent variables

Observed COVID-19 case fatality and infection rates were the dependent variables of this study, in which the former one was defined as the proportion of people who die from COVID-19 among all individuals diagnosed with COVID-19 and considered per 10,000 COVID-19 positive cases. COVID-19 infection rate was defined as the proportion of people who were COVID-19 positive among susceptible population (considered as the total population to calculate the infection rate per 10,000 population).

### Explanatory variables

#### UHC for service coverage index and GHS index

UHC for service coverage index and overall GHS index were the core explanatory variables. They were primary variables of interest for this study. Universal health service coverage index data were obtained from the WHO Global Health Observatory Repository. The index reflects the extent to which people receive healthcare services they need. Data used to calculate the index were obtained from different databases. It represents coverage for essential health services (based on tracer interventions that include reproductive, maternal, newborn and child health, infectious diseases, non-communicable diseases and service capacity and access).It is presented on a scale of 0 to 100 with higher values indicating greater coverage. The data for 2017 were used for this study [29].

The 2019 Global Health Security Index (GHS Index) is designed as the first comprehensive assessment and benchmarking of health security and capabilities across 195 countries in order to assess the country’s ability to avert and mitigate infectious disease outbreaks that can lead to international epidemics and pandemics. The report summarizes the results of the first Global Health Security Index, which includes a component to prevent, detect, and respond, health, norms and risk to infectious disease threats in the country. The GHS Index is a project of the Nuclear Threat Initiative (NTI) and the Johns Hopkins Center for Health Security (JHU) and was developed with The Economist Intelligence Unit (EIU) [25].

#### Additional control variables

In addition to the UHC for service coverage index and GHS index, there are other variables that determine observed COVID-19 case fatality and infection rates. In order to assess effects of these variables on COVID-19 case fatality and infection rates, the study included in the analysis as control variables set. Based on the previous research results, we selected the following indicators as a covariate to assess social risk factors for susceptibility and acquire infection and case fatality rate. These included the median age of the national population, population density per square kilometer; the prevalence of obesity among adults aged 18 + years expressed as a body mass index (BMI) ≥ 30 kg/m^2^, percentage who currently use tobacco, the ambient and household air pollution attributable death rate (per 100,000 population), and disease specific mortality of chronic obstructive pulmonary disease (COPD).

The details description of the explanatory variables such as of the UHC for service coverage and GHS component as well as other covariates is attached in the supplementary annex ( Additional file 2 ).

### Statistical analysis

A cross-sectional data on the total number of COVID-19 cases and deaths reported as of June 26, 2021 were used to estimate association of the case fatality and infection rates with that of health, comorbidities and country level characteristics on this date. The data collected in CSV format was exported into Microsoft excel 2017 computer program package. Descriptive data were reported for categorical variables and expressed as frequencies and percentages, while continuous variables were expressed in terms of arithmetic means and standard deviations (SDs) as well as median, IQR and minimum and maximum values. Employing Structural Equation Modeling (SEM) with maximum likelihood estimation method, the study examined the relationship between independent and dependent variables. Moreover, the study perform path analysis to assess the direct and indirect effect of the key variables on the two dependent variables. Path coefficients of the key parameters and their significance levels were determined from the path way regression after running SEM.

Overall model fitness test have been made using different statistical indices. The indices were standardized root mean square residual (SRMR) (threshold value < 0.08 indicate adequate/good fit); coefficient of determination (CD) (similar to the R-squared value, ranging 0–1, values closer to 1 indicate good fit) and maximum likelihood estimation (MLE) method (values of p-value > 0.05 indicate a good fit), comparative fit index (CFI) > 0.90 [30], Tucker–Lewis Index (TLI) > 0.90, chi-square test (χ2/ degrees of freedom (df) ratio < 5 [31], Pclose > 0.05, root mean square error of approximation (RMSEA) ≤ 0.08 [32] were used as standard criterion to determine model fitness. The overall significance level was tested at *P* < 0.05. All statistical analyses were performed using Stata (Statistical Data Analysis Package version 14.0 SE, College Station, TX–USA).

**Fig. 1:**
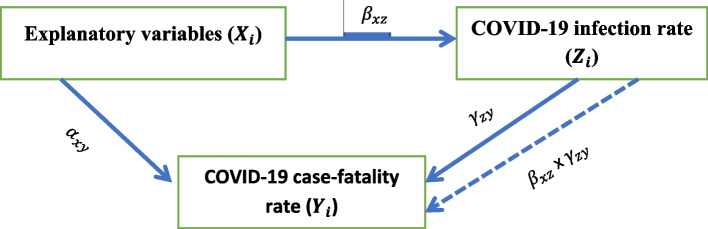
Shows the effect of explanatory variables on COVID-19 infection rate and COVID -19 fatality rate

Figure 1 shows a path diagram for the causal relationships between the three variables such as the Explanatory (${\varvec{X}}_{\varvec{i}}$), and the dependent ones that comprise the dependent (Covid-19 case fatality rate (${\varvec{Y}}_{\varvec{i}}$) and endogenous ( COVID-19 Infection rate (${\varvec{Z}}_{\varvec{i}}$) variables. The explanatory variables (${\varvec{X}}_{\varvec{i}}$) are exogenous, which comprise universal health coverage index, prevalence to other diseases, population density, median age of the population and related ones. In the study, the three groups of variables are assumed to be all observed, then the formulation considered rectangles (not circles) format of presenting the variables. In the figure the solid lines show direct effect, while the broken ones refer mediation effect of the explanatory variables through the endogenous variable.

Based on Bollen and Pearl (2013) [33] and Imai et al. (2010) [34] the study assume that the error terms (${e}_{zi}$and ${e}_{yi}$ *)* in the following two equation are uncorrelated, which is an important and crucial assumption for causal inference to undertake mediation analysis. Moreover, the study also assumed multivariate normality for the error terms, which is a necessary and underlying condition of defining direct, indirect and total effects. Note that, the following two structural equations are linked together through the endogenous variable and inference about them is simultaneous, unlike two independent standard regression equations.

Based on the above graph the **direct effect** is the pathway (**the solid line**) from the exogenous and endogenous variables to the outcome while controlling for the mediator. Thus, in the path diagrams, and is the direct effects on the endogenous and outcome variables, respectively. Moreover, the figure shows the **indirect effect**, which describes the pathway from the exogenous variable to the outcome variable (COVID-19 case-fatality rate) through the mediator or the endogenous variable that is infection rate. This path is represented through the product of, and. Given this, the **total effect** of the explanatory variables on the outcome variable could be the sum of the direct and indirect effects that is $\alpha_{xy}$+$\beta_{xz}$ $Y_{zy}$. Thus, the regression equation could be formulated as:-.

$$Z_i=\beta_0+\beta_{xz}X_i+\varepsilon_{zi}\;,$$

$$Y_i=\alpha_0+\alpha_{xy}X_{i+}Y_{zy}Z_i+\varepsilon_{yi}$$

Based on significance level of the coefficients ${a}_{xy}$, and ${Y}_{zy}$, the study could evaluate interaction of the explanatory and outcome variables. If value of the coefficient is zero (${Y}_{zy}=0$), then two variables are not interdependent. Hence, the study should not consider mediation. Thus, mediation effect analysis could be made if the coefficient is different from zero ($({Y}_{zy} \not= 0)$). Full mediation, which consider the direct, indirect and total effects could be made and multiple direction effect of the potential variables have been examined [35].

## Results

The descriptive statistics of all variables were presented in Table 1. Briefly, in terms of percentage who currently use tobacco, mean and median are the same, indicating that 50% of the samples are above the average level of the variable values. Besides, the mean is greater than the median in the entire variable except current tobacco use, which reveals that more than 50% of the sample does not reach the average level of each of the variables. The average UHC for service coverage index is 48.07 (out of a possible 100) while it is 31.07 (out of a possible 100) for overall GHS index.

**Table 1:** Descriptive statistics

Variables	Obs	Mean	SD	Min	Max	Median	IQR
Observed COVID-19 case fatality rate (CFR)	54	2.16	1.44	0.15	7.52	1.65	1.96
COVID-19 infection rate	54	951.5	2283.6	0.83	15277.02	233.46	506.92
Median age of the national population	54	21.08	4.90	15.1	37.4	19.4	4.1
Overall GHS index	54	31.07	7.79	16.2	54.8	30.65	9.3
Population density per square kilometer	54	107.22	135.5	3.0	626	61.5	90
Percentage who currently use tobacco	54	13.60	5.15	4.25	27.2	13.60	5.7
Disease specific mortality of Chronic Obstructive Pulmonary Disease (COPD).	54	6.82	3.08	2.74	22.48	6.46	3.33
UHC for service coverage index	54	48.07	12.41	25	78	45.0	15
Prevalence of obesity among adults, BMI ≥ 30 kg/m2	54	10.53	7.06	3.6	31.8	8.2	5.9
Ambient and household air pollution attributable death rate (per 100,000 population)	54	89.38	33.35	39.89	180.9	82.71	44.18

*UHC *Universal health coverage (UHC), *GHS index *Global Health Security Index;

The results of the Structural Equation Modeling (SEM) were indicated in Table 2 using estimates of path coefficients and standard errors with their respective *p* values. The overall goodness of fit of the structural model predicting COVID-19 case-fatality rate and COVID-19 infection rate was adequate based on the standard fit criteria ( Likelihood ratio (x2) (df) = 2.839(0.585); SRMR = 0.020 ;CD (R2) = 0.684 ;RMSEA = 0.000; Pclose = 0.646 ; CFI = 1.000 and TLI = 1.097). In Table 2, the pathway among COVID-19 case-fatality rate and median age of the national population (β = -0.1244, [95% CI: -0.24, -0.01], *P* = 0.031 ); COVID-19 infection rate (β = -0.370, [95% CI: -0.66, -0.08], *P* = 0.012 ); and prevalence of obesity among adults aged 18 + years (β = 0.128, [95% CI: 0.06,0.20], *P* = 0.0001) were statistically significant. Nevertheless, there was no significant relationship between COVID-19 case-fatality rate and prevalence to chronic obstructive pulmonary disease (COPD); UHC for service coverage index as well as overall GHS index (see Table 2).

**Table 2:** Unstandardized Path coefficient estimates of predicting COVID-19 Infection rate and case-fatality rate

Variables	Path coefficient	Std. Err.	z -statistics	p-value	[95% Conf. Interval]
**COVID-19 case-fatality rate**					
lnInfection rate	-0.370	0.147	-2.52	0.012	-0.66, -0.08
Median age of the national population	-0.124	0.058	-2.16	0.031	-0.24, -0.01
Percentage who currently use tobacco	0.065	0.037	1.73	0.085	-0.01, 0.14
Disease specific mortality of Chronic Obstructive Pulmonary Disease (COPD).	-0.009	0.057	-0.17	0.868	-0.12,0.10
Overall GHS index	0.002	0.022	0.11	0.913	-0.04, 0.05
Prevalence of obesity among adults expressed as BMI ≥ 30 kg/m2	0.128	0.037	3.49	0.000	0.06,0.20
UHC for service coverage index	0.004	0.026	0.17	0.868	-0.05,0.06
Constant	4.386	1.114	3.94	0.000	2.20,6.57
**COVID-19 lnInfection rate**					
Median age of the national population	0.118*	0.052	2.26	0.024	0.02,0.22
Overall GHS index	-0.024	0.019	-1.25	0.212	-0.06, 0.01
Population density per square kilometer	-0.003	0.001	-2.40	0.016	-0.0058, -0.00059
Prevalence of obesity among adults expressed as BMI ≥ 30 kg/m2	-0.057	0.040	-1.41	0.158	-0.14,0.02
Air pollution attributable death rate (per 100,000 population)	0.0005	0.005	0.09	0.932	-0.01, 0.01
UHC for service coverage index	0.089	0.027	3.41	0.001	0.04,0.14
Constant	0.444	1.564	0.28	0.776	-2.62, 3.51
Universal Health Coverage Index → Global Health Security Index	0.441*	0.208	2.11	0.034	0.03,0.85
Constant	34.389	6.674	5.15	0.000	21.30,47.46
var(e.observedcasefatalityra ~ r)	1.404	0.270			0.96,2.05
var(e.infectionrateper100000)	1.115	0.214			0.76,1.63
var(e.UHC_s ~ e	139.65	26.88			95.77,203.64
**Goodness of fit**					
Likelihood ratio(x2)(df)	2.839(0.585)				
SRMR	0.020				
CD(R^2^)	0.684				
RMSEA	0.000				
pclose	0.646				
CFI	1.000				
TLI	1.097				

*UHC *Universal health coverage (UHC), *GHS index *Global Health Security Index, *SRMR *Standardized root mean squared residual, *CD *Coefficient of determination, *RMSEA *Root mean squared error of approximation, *CFI *Comparative fit index, *TLI *Tucker-Lewis index, *Pclose *Probability RMSEA < = 0.05 ; * Significant at *P* < 0.05

**Fig. 2:**
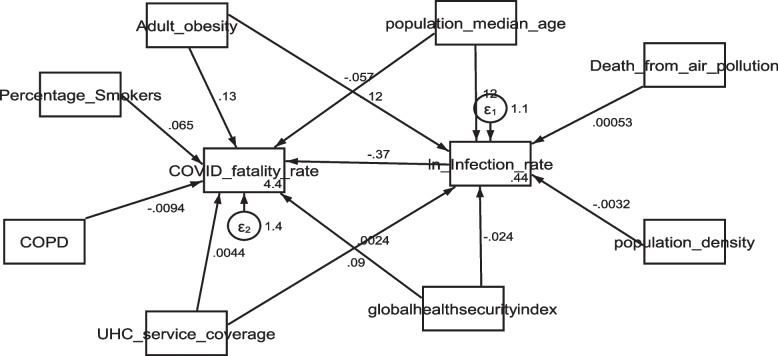
Path analysis diagram with standardized estimates illustrating the total effects of explanatory parameters and COVID-19 infection rate and COVID -19 fatality rate

In addition, this study also showed the pathway between COVID-19 infection rate and median age of the national population (β = 0.118, [95% CI: 0.02,0.22 ], *P* = 0.024); population density per square kilometer, in which their relationship was statistically significant (β = -0.003, [95% CI: -0.0058, -0.00059], *P* = 0.016) and UHC for service coverage index (β = 0.089, [95% CI: 0.04,0.14], *P* = 0.001). However, there was no significant relationship between COVID-19 infection rate and the ambient and household air pollution attributable death rate, prevalence of obesity among adults expressed as well as overall GHS index (Table 2). Figure 2 shows the path analysis diagram with standardized estimates illustrating the total effects of explanatory parameters and COVID-19 infection rate and COVID − 19 fatality rate .

The total effect of GHS index on COVID-19 infection rate was − 0.024. The direct component of this total effect was − 0.024, in other words, -0.024/-0.024 = 1 or 100% of the effect of GHS index on COVID-19 infection rate is direct. (Tables 3 and 5). In the same fashion, the total effect of GHS index on COVID-19 case-fatality rate was 0.011. The direct component of this total effect was 0.002, or in other words, 0.002 /0.011 = 0.18 or 18% of the effect of GHS index on COVID-19 case-fatality rate is direct (Tables 3 and 5). By contrast, the indirect effect was 0.009, so that 0.8182 or 81.82% of the effect of GHS index was partially mediated by UHS index (Tables 4 and 5).

**Table 3:** Estimation of direct effect of universal health coverage index and global health security index on COVID-19 Infection rate in Africa

Variables	Path coefficient	Std. Err.	z -statistics	p-value	[95% Conf. Interval]
**COVID-19 case-fatality rate**					
lnInfection rate	-0.370	0.147	-2.52	0.012	-0.66, -0.08
Median age of the national population	-0.124	0.058	-2.16	0.031	-0.24,-0.01
Percentage who currently use tobacco	0.065	0.037	1.73	0.085	-0.01, 0.14
Disease specific mortality of Chronic Obstructive Pulmonary Disease (COPD).	-0.009	0.057	-0.17	0.868	-0.12, 0.10
Overall GHS index	0.002	0.022	0.11	0.913	-0.04, 0.05
Prevalence of obesity among adults expressed as BMI ≥ 30 kg/m2	0.128	0.037	3.49	0.000	0.06,0.20
UHC for service coverage index	0.004	0.026	0.17	0.868	-0.05, 0.06
Constant	4.386	1.114	3.94	0.000	2.20, 6.57
**COVID-19 lnInfection rate**					
Median age of the national population	0.118*	0.052	2.26	0.024	0.02, 0.22
Overall GHS index	-0.024	0.019	-1.25	0.212	-0.06,0.01
Population density per square kilometer	-0.003	0.001	-2.40	0.016	-0.0058, -0.00059
Prevalence of obesity among adults expressed as BMI ≥ 30 kg/m2	-0.057*	0.040	-1.41	0.158	-0.14,0.02
Air pollution attributable death rate (per 100,000 population)	0.0005	0.005	0.09	0.932	-0.01, 0.01
UHC for service coverage index	0.089	0.027	3.41	0.001	0.04,0.14
Constant	0.444	1.564	0.28	0.776	-2.62,3.51

*UHC *Universal health coverage (UHC), *GHS index *Global Health Security Index, *SRMR *Standardized root mean squared residual, *CD *Coefficient of determination * Significant at *P* < 0.05

**Table 4:** Estimation of indirect effect of universal health coverage index and global health security index on COVID-19 Infection rate in Africa

Variables	Path coefficient	OIM Std. Err.	z -statistics	p-value P>|z|	[95% Conf. Interval]
**COVID-19 case-fatality rate**					
lnInfection rate	-	no path			
Median age of the national population	-0.044	0.026	-1.68	0.093	-0.09,0.01
Percentage who currently use tobacco	-	no path			
Disease specific mortality of Chronic Obstructive Pulmonary Disease (COPD).	-	no path			
Overall GHS index	0.009	0.008	1.12	0.264	-0.01, 0.02
Prevalence of obesity among adults expressed as BMI ≥ 30 kg/m2	0.021	0.017	1.23	0.219	-0.01,0.05
UHC for service coverage index	-0.033	0.016	-2.02	0.043	-0.06,-0.001
Ambient and household air pollution attributable death rate (per 100,000 population)	-0.002	0.002	-0.09	0.932	-0.005,0.004
Population density per square kilometer	0.001	0.0007	1.74	0.082	-0.00015,0.0025
**COVID-19 lnInfection rate**					
Median age of the national population	-	no path			
Overall GHS index	-	no path			
Population density per square kilometer	-	no path			
Prevalence of obesity among adults expressed as BMI ≥ 30 kg/m2	-	no path			
Air pollution attributable death rate (per 100,000 population)	-	no path			
UHC for service coverage index	-	no path			

*UHC *Universal health coverage (UHC), *GHS index *Global Health Security Index, *SRMR *Standardized root mean squared residual, *CD *Coefficient of determination * Significant at *P* < 0.05

**Table 5:** Estimation of total effect of universal health coverage index and global health security index on COVID-19 Infection rate in Africa

Variables	Path coefficient	OIM Std. Err.	z -statistics	*p*-value P>|z|	[95% Conf. Interval]
**COVID-19 case-fatality rate**					
lnInfection rate	-0.370	0.147	-2.52	0.012	-0.66,-0.08
Median age of the national population	-0.168	0.060	-2.79	0.005	-0.28, -0.05
Percentage who currently use tobacco	0.065	0.037	1.73	0.085	-0.01,0.14
Disease specific mortality of Chronic Obstructive Pulmonary Disease (COPD).	-0.009	0.057	-0.17	0.868	-0.12,0.10
Overall GHS index	0.011	0.023	0.50	0.620	-0.03,0.06
Prevalence of obesity among adults expressed as BMI ≥ 30 kg/m2	0.149	0.040	3.70	0.000	0.07,0.23
UHC for service coverage index	-0.029	0.026	-1.11	0.268	-0.08,0.02
Ambient and household air pollution attributable death rate (per 100,000 population)	-0.0001	0.002	-0.09	0.932	-0.0047,0.0043
Population density per square kilometer	0.0012	0.0007	1.74	0.082	-0.00015,0.00252
**COVID-19 lnInfection rate**					
Median age of the national population	0.118	0.052	2.26	0.024	0.01,0.22
Overall GHS index	-0.024	0.019	-1.25	0.212	-0.06,0.01
Population density per square kilometer	-0.003	0.001	-2.40	0.016	-0.006,-0.001
Prevalence of obesity among adults expressed as BMI ≥ 30 kg/m2	-0.057	0.040	-1.41	0.158	-0.14,0.02
Air pollution attributable death rate (per 100,000 population)	0.0005	0.006	0.09	0.932	-0.01,0.01
UHC for service coverage index	0.089	0.026	3.41	0.001	0.04,0.14

*UHC *Universal health coverage (UHC), *GHS index *Global Health Security Index, *SRMR *Standardized root mean squared residual, *CD *Coefficient of determination * Significant at *P* < 0.05

## Discussion

In this study, the authors considered publicly available data to assess the factors that aggravate COVID-19 infection and case-fatality rates in Africa by considering different explanatory variables. The finding revealed that both, UHC and GHS do not emerge to protect against COVID-19-related case fatality rate, however, UHC service coverage appear to protect against COVID-19 infection rate. Some variables such as median age of the national population, COVID-19 infection rate; and prevalence of obesity among adults aged 18 + years significantly aggravated the COVID-19 case-fatality rate. In addition, COVID-19 infection rate was associated with population density, UHC for service coverage index and median age of the national population.

As indicated in several studies older age was associated with a higher COVID-19 case-fatality rate wherein the natural experimental study in South Korea identified that older age was significantly associated with higher odds of COVID-19 case fatality [36]. Other studies consistently documented that older age is a major risk factor for mortality [37–42]. One study carried out in western countries (Europe, USA and Canada) mentioned that each 5% point increment of this older age group among confirmed SARS-CoV-2 cases was associated with an increase in case fatality rate RT-PCR CFR of 2.5% points [43] and in Italy, a substantial increase in the RT-PCR CFR which is attributable to increasing age-specific case-fatality [42, 44]. A study done in sub-Saharan Africa showed, the mean RT-PCR CFR estimates increased with age, and the sex-specific RT-PCR CFR differences being significant among those aged 40 years or more [45]. Countries’ mortality outcome was strongly predicted by the percentage of the population aged 65 and above [46]. The fact that SARS-CoV-2 infection caused a severe and rapid progression in older patients who required faster hospitalization, presented more serious symptoms on admission, and had a worse clinical course [39, 47] and age has emerged as a major factor to predict the severity of the disease and mortality rates are significantly higher in elderly patients [48, 49].

Another factor illustrated in our study for increase RT-PCR CFR was infection rate increment. Similar findings were reported previously and argued that asymptomatic SARS-CoV-2 infection were more common in young people, and fatal cases were more common in older adults [50], in which in turn affect the case fatality rate. Previous study carried out using ecological analysis of 65 countries found that the cumulative number of infected patients in each country was directly and positively associated with the case fatality rate [51].

In this study, there is relationship has been observed between prevalence of obesity among adults aged 18 + years and COVID-19 case-fatality rate. The finding was consistent with other studies, which identified that obesity has emerged as a novel risk factor for hospitalization, greater risk of severe disease and death due to COVID-19 [48, 52–54]. Across 168 countries for which data were available, higher obesity prevalence was associated with increased COVID-19 mortality and prevalence rates [55]. Covid-19 death rates are 10 times higher in countries where more than half of the adult population is classified as overweight [56] and evidence of a significant impact of overweight/obesity prevalence on the increase in COVID-19 morbidity/mortality [57]. In retrospective observational cohort study, included patients seen at an Ochsner Health facility based in New Orleans, obesity was associated with an increased odds of hospital admission in United State of Louisiana [58]. The presence of any coexisting illness was more common among patients with severe disease than among those with non-severe disease (38.7% vs. 21.0%) in a study carried out with COVID-19 from 552 hospitals in 30 provinces in China [38].

Epidemiological studies reported that obesity is associated with a higher rate of mortality in patients with COVID-19 [58–60]. This is due to the fact that obesity is linked to increased comorbidity like diabetes, hypertension, and heart disease [59] as well as obesity is inversely associated with lung function in adults, but central fat distribution appears to have a stronger relationship with respiratory mechanics and independent of the degree of physical activity and aerobic fitness [61]. In the same context, the burden of severe COVID-19 outcomes was highest in countries with the prevalence of pre-existing health risk factors including cancer, diabetes, air pollution and obesity was high [7, 49]. In addition to this, one study in South Korea reported that there was a socioeconomic disparity in COVID-19 prevalence and fatality despite UHC (i.e. South Korea has achieved perfect UHC—all Koreans receive UHC regardless of their socioeconomic status). However, disparities in fatality were not due to socioeconomic status, but due to the poor underlying health conditions of the people [62]. One study showed that when countries were compared based on the presence of UHC or non-UHC, countries with UHC had 13,789,891 cases, which was about 37.6% of all the cases reported globally. However, the countries with UHC experienced a much higher proportion of fatalities (56.4%). The RT-PCR CFR of countries with UHC (10.5%) was more than double that of countries without UHC (4.9%) and UHC does not appear to protect against mortality in a pandemic environment such as with COVID-19 [63], which might be attributed to a mixture of the effects of several types of UHC such as coverage and preparedness of the healthcare system.

As examined by several other studies, the spread of the infection depends on a lot of social and economic factors such as population density, healthcare system level, mortality rate of non-communicable chronic diseases (NCD) [57, 64, 65]. The issue of population density related with infection rate is relied on other parameters and socio-demographic ingredients. Population density has been used as a surrogate measure of social distancing capacity and potential indicator of infection spread. Several studies showed that SARS-CoV-2 transmission is potentially more likely to occur among cities with higher population densities [66–69]. Recent evidence reported that vulnerable people often have more underlying comorbidities and they are more exposed to hazardous environments, such as densely populated neighborhoods, occupations, and housing conditions [70] and more likely to get infected, less likely to be treated properly, and as a result, more likely to die because of COVID-19 [71] as well as experience delayed diagnostic testing and insufficient treatment induced by a lack of health insurance, related to UHC, leading to poor prognosis and negative treatment outcome [72]. With high caseloads looming, many countries could face multiple challenges to ultimately controlling the virus, especially in light of societal realities, such as densely populated settlements [9].

Globalization, settlement and population characteristics, and variables related to high human mobility lead to greater reported disease diffusion, mainly from more- to less-developed countries and regions, and hierarchical diffusion from countries with higher population and density [73]. Apparently, residence in a low-income area was associated with an increased odds of hospital admission [58]. Empirical analysis demonstrated that counties with greater population density have greater rates of transmission (R0) of SARS-CoV-2, likely due to increased contact rates in areas with greater density necessary for disease transmission [69]. Population density and more urbanized populations, have influenced the arrival of the first case and the within-country speed of the epidemic especially in the context of the whole African continent [74]. Evidences suggested that dense urban populations are vulnerable to higher rates of crowding and social interactions, in which more likely increases the risk for the spread of directly transmitted aerial infectious agents in particular [73].

Our results are consistent with recent studies that reported median age of the population is associated with increased COVID-19 infection rate. This is evidenced that though the countries having higher percentage of aged populations are more prone to be affected by the spread of virus [64]. SARS-CoV-2 infection burden was highest in countries that had larger economies or greater capacity for case detection and the burden of severe COVID-19 outcomes was highest in countries with older populations [49]. Population density and population characteristics such as total population, older populations, and household size are strong predictors in early weeks but have a muted impact over time on reported COVID-19 diffusion [73] and spread across the community. There were some evidences and consistent findings that link between UHC for service coverage index and COVID-19 infection rate. Data from 96 countries reported that strengthening national health security capacities would in turn contribute to the achievement of UHC that would further guide for better implementation of International Health Regulations (IHR) [75]. Similar findings were indicated that the epidemic of COVID-19 in China demonstrated the benefit of strong health systems to mitigate the impact of epidemics. There were higher case-fatality rates in Hubei (about 2·9% on average), which had weaker health systems capacity, than the other provinces of China (about 0·7% on average) [76]. In similar way, higher numbers of infections in a given population can be considered an indirect indicator of a heavier health-care burden, drawing the mortality against the incidence of COVID-19 showed a significant positive correlation, which indicates that mortality is correlated with health-care burden and healthcare system performance [71]. In addition to this, an Italian regional data showed a strong correlation between RT-PCR CFR and COVID-19 incidence [77], which is also in concordance with our finding that postulate the relationship of COVID-19 case-fatality with infection rates. Likewise, the burden of severe COVID-19 outcomes was highest in countries with lower healthcare capacity [49]. Increasing literatures in the past highlighted that countries outside China most severely hit by the first wave of the pandemic have well-established healthcare systems [78]. The paradoxical issue is that the lower numbers of reported COVID-19 cases and deaths in African countries relative to the rest of the world represented lower rates of transmission due to control measures, fewer symptomatic infections due to younger populations, or simply weaker detection capacities than in high-resource settings.

Overall, from the perspective of global hath security and its index ((GHSI) and preparedness and response, Joint External Evaluation (JEE) and States Parties Self-Assessment Annual Reporting (SPAR)) have been adopted to track the IHR implementation stage in each country [75], in which case, JEE was associated with the UHC score (*r* = 0.85, *p* < 0.001) and SPAR was also associated with the UHC service coverage index (*r* = 0.81, *p* < 0.001). The JEE and SPAR scores showed a significant positive correlation with the UHC service coverage index after adjusting for several confounding variables, which is in accordance with our finding [75]. In similar fashion, both GHSI (*r* = 0.31) and JEE (*r* = 0.37) had a weak correlation with countries’ COVID-19-related mortality outcome, however overall GHSI score and JEE were not significant predictors [46]. Despite the finding that the GHS did not have an association with COVID-19 mortality and infection rate in our finding, the bottom line is preparedness and response to public health crises requires strengthening health systems and inter-sectoral approaches, which in turn contribute to improving global health security and achieving UHC [75].

In our study, we have some limitations observed and anticipated for generalizability of the findings in this study. Reporting bias, as the study results are based on the data reported by each country, and data have been extracted from various institutions and websites. Detection and time bias might have also affected the results because the pathway for the GHS with infection and case fatality was not picked in our study, there might be other variables to consider in the line. Quality of the data is another potential weakness regarding the aggregate of data collated from countries due to the nature of the study design-ecological study. Other concern that should be raised as a limitation is the use of sero-excess infection fatality ratio (IFR), when the numbers of excess deaths are used as a proxy for COVID-19 deaths and the numbers of infections are inferred via serological surveys, a sero-excess IFR is obtained. However, the sero-excess IFR is the best in principle, but may not be feasible at present owing to data availability [79].

## Conclusions

In conclusion, the study assessed that UHC for service coverage index, and median age of the national population and population density have significant effect on COVID-19 infection rate in African countries. Similarly, COVID-19 infection rate, median age of the national population and prevalence of obesity among adults aged 18 + years have significant effect of aggravating COVID-19 case-fatality rate among countries in the continent. The finding also indicated that both, UHC and GHS do not emerge to protect against COVID-19-related case fatality rate, however, UHC service coverage appear to protect against COVID-19 infection rate.

Thus, the study recommended that the citizen of the continent should have strong prevention measure where there is high population density and obesity (likely in urban areas) and maybe strong/ tailored prevention and treatment approach for those obese as they likely to die of COVID-19. Having strong and good UHC status, one of the many measures of healthcare access, plays a significant role in determining the severity of the spread of infectious disease such as COVID-19. Additionally, institutional and social measures to promote development and eliminate poverty by improved the socio-economic status /living conditions has to be streamed. Lastly, country level determinants can be addressed by the respective government measures to control the spread of the infection and reduce mortality across African continent. Future studies in the area of COVID-19 epidemiology and understanding the burden of the disease with strong study design with other variables in addition to the variables stated in the current study should be considered as arecommendations.

## Supplementary Information

**Additional file 1: Appendix 1.** List of Africa countries included in this study

**Additional file 2: Appendix 2.** Explanatory variables, descriptions and sources

## Data Availability

The data analysed during this study are included in this article and are available from the corresponding author upon reasonable request.

## Abbreviations

CFR: Case-Fatality Rates
COPD: Chronic Obstructive Pulmonary Disease
COVID-19: Coronavirus Disease 2019
GHS: Global Health Security
IHR: International Health Regulations
JEE: Joint External Evaluation
LMIC: Low Middle Income Countries
PHC: Primary Health Care
RT-PCR CFR: Reverse-Transcriptase‐Polymerase Chain Reaction
SARS-CoV-2: Severe Acute Respiratory Syndrome Coronavirus 2
SEM: Structural Equation Modeling
UHC: Universal Health Coverage
WHO: World Health Organization

## References

[1] World Health Organization. WHO Director-General’s opening remarks at the media briefing on COVID-19–11 March 2020. WHO https://www.who.int/dg/speeches/detail/who-director-general-s-opening-remarks-at-the-media-briefing-on-covid-19---11-march-2020 2021.

[2] Cucinotta D, Vanelli M (2020). WHO declares COVID-19 a pandemic. Acta bio-medica: Atenei Parmensis.

[3] Tay MZ, Poh CM, Rénia L, MacAry PA, Ng LFP (2020). The trinity of COVID-19: immunity, inflammation and intervention. Nat Rev Immunol.

[4] Abbey EJ, Khalifa BAA, Oduwole MO, Ayeh SK, Nudotor RD, Salia EL, Lasisi O, Bennett S, Yusuf HE, Agwu AL (2020). The Global Health Security Index is not predictive of coronavirus pandemic responses among Organization for Economic Cooperation and Development countries. PLoS ONE.

[5] WHO. : Weekly epidemiological update on COVID-19–4 May 2022, World Health Organization(WHO), Edition 90(Available at https://www.who.int/publications/m/item/weekly-epidemiological-update-on-covid-19---4-may-2022). 2022.

[6] Estimating excess mortality (2022). Due to the COVID-19 pandemic: a systematic analysis of COVID-19-related mortality, 2020-21. Lancet (London England).

[7] Okpokoro E, Igbinomwanhia V, Jedy-Agba E, Kayode G, Onyemata EJ, Abimiku AL. Ecologic correlation between underlying population level morbidities and COVID-19 case fatality rate among countries infected with SARS-CoV-2. medRxiv. 2020:2020–04.

[8] Vasishtha G, Mohanty SK, Mishra US, Dubey M, Sahoo U (2021). Impact of COVID-19 infection on life expectancy, premature mortality, and DALY in Maharashtra, India. BMC Infect Dis.

[9] Lal A, Erondu NA, Heymann DL, Gitahi G, Yates R (2021). Fragmented health systems in COVID-19: rectifying the misalignment between global health security and universal health coverage. Lancet (London England).

[10] Saulnier DD, Blanchet K, Canila C, Muñoz DC, Dal Zennaro L, de Savigny D, Durski KN, Garcia F, Grimm PY, Kwamie A, Maceira D (2021). A health systems resilience research agenda: moving from concept to practice. BMJ Global Health..

[11] 2019 Global Health Security Index. https://www.ghsindex.org, accessed on Oct 2021. 2021.

[12] Wenham C, Katz R, Birungi C, Boden L, Eccleston-Turner M, Gostin L, Guinto R, Hellowell M, Onarheim KH, Hutton J (2019). Global health security and universal health coverage: from a marriage of convenience to a strategic, effective partnership. BMJ global health.

[13] Erondu NA, Martin J, Marten R, Ooms G, Yates R, Heymann DL (2018). Building the case for embedding global health security into universal health coverage: a proposal for a unified health system that includes public health. Lancet (London England).

[14] Kutzin J, Sparkes SP (2016). Health systems strengthening, universal health coverage, health security and resilience. Bull World Health Organ.

[15] Karamagi HC, Tumusiime P, Titi-Ofei R, Droti B, Kipruto H, Nabyonga-Orem J, Seydi AB, Zawaira F, Schmets G, Cabore JW (2021). Towards universal health coverage in the WHO African Region: assessing health system functionality, incorporating lessons from COVID-19. BMJ global health..

[16] Kruk ME, Myers M, Varpilah ST, Dahn BT (2015). What is a resilient health system? Lessons from Ebola. Lancet (London England).

[17] Heymann DL, Chen L, Takemi K, Fidler DP, Tappero JW, Thomas MJ, Kenyon TA, Frieden TR, Yach D, Nishtar S (2015). Global health security: the wider lessons from the west african Ebola virus disease epidemic. Lancet (London England).

[18] Patel MS, Phillips CB (2015). Health security and political and economic determinants of Ebola. Lancet (London England).

[19] Bali S, Dhatt R, Lal A, Jama A, Van Daalen K, Sridhar D (2020). Off the back burner: diverse and gender-inclusive decision-making for COVID-19 response and recovery. BMJ Global Health..

[20] Elebesunu EE, Oke GI, Adebisi YA, Nsofor IM (2021). COVID-19 calls for health systems strengthening in Africa: A case of Nigeria. Int J Health Plann Manage..

[21] Amos OA, Adebisi YA, Bamisaiye A, Olayemi AH, Ilesanmi EB, Micheal AI, Ekpenyong A, Lucero-Prisno DE (2021). COVID-19 and progress towards achieving universal health coverage in Africa: a case of Nigeria. Int J Health Plann Manag.

[22] Gyeltshen D, Musa SS, Amesho JN, Ewelike SC, Bayoh AVS, Al-Sammour C, Camua AA, Lin X, Lowe M, Ahmadi A (2021). COVID-19: a novel burden on the fragile health system of Angola. J global health.

[23] Hasan MM, Costa A, Xenophontos E, Mohanan P, Bassey EE, Ahmad S, Essar MY (2021). Lassa fever and COVID-19 in Africa: a double crisis on the fragile health system. J Med Virol.

[24] Worldometer. Coronavirus Update (Live): Cases and Deaths from COVID-19 Virus Pandemic [Internet]. Worldometers. 2021. Available from: https://www.worldometers.info/coronavirus/. 2021.

[25] GHS: The Global Health Security Index(GHS) Index. Accessed on. July 2022; Available from https://www.ghsindex.org/ 2022.

[26] Our World in Data. Our world in Data website, University of Oxford, Oxford, England, https://ourworldindata.org. 2022.

[27] WHO: The Global Health Observatory (database). World Health Organization. Geneva; 2022. http://www.who.int/gho/database/en/. (accessed on 04 March 2022).

[28] World Bank. World Development Indicators (database), DataBank, World Bank Group, Washington DC. https://databank.worldbank.org/source/world-development-indicators (accessed on 04 March 2022) 2022.

[29] Hogan DR, Stevens GA, Hosseinpoor AR, Boerma T (2018). Monitoring universal health coverage within the Sustainable Development Goals: development and baseline data for an index of essential health services. The Lancet Global health.

[30] Hu Lt, Bentler PM (1999). Cutoff criteria for fit indexes in covariance structure analysis: conventional criteria versus new alternatives. Struct equation modeling: multidisciplinary J.

[31] Schumacker RE, Lomax RG. A beginner's guide to structural equation modeling. psychology press; 2004.

[32] Browne MW, Cudeck R. Alternative ways of assessing model fit. Sociological methods & research. 1992;21(2):230–58.

[33] Bollen KA, Pearl J. Eight myths about causality and structural equation models. Handb causal Anal social Res. Dordrecht: Springer Netherlands; 2013. p. 301–28.

[34] Imai K, Keele L, Tingley D (2010). A general approach to causal mediation analysis. Psychol Methods.

[35] Bollen KA, Stine R (1990). Direct and indirect effects: Classical and bootstrap estimates of variability. Sociol Methodol..

[36] Lee H, Lee JR, Jung H, Lee JY. Factors associated with incidence, mortality, and case fatality of COVID-19: A natural experimental study in South Korea. Mortality, and Case Fatality of COVID-19: A Natural Experimental Study in South Korea. 2020.

[37] Huang C, Wang Y, Li X, Ren L, Zhao J, Hu Y, Zhang L, Fan G, Xu J, Gu X (2020). Clinical features of patients infected with 2019 novel coronavirus in Wuhan, China. The lancet.

[38] Guan W-j, Ni Z-y, Hu Y, Liang W-h, Ou C-q, He J-x, Liu L, Shan H (2020). Lei C-l, Hui DS: clinical characteristics of coronavirus disease 2019 in China. N Engl J Med.

[39] Casas-Deza D, Bernal-Monterde V, Aranda-Alonso AN, Montil-Miguel E, Julián-Gomara AB, Letona-Giménez L, Arbones-Mainar JM (2021). Age-related mortality in 61,993 confirmed COVID-19 cases over three epidemic waves in Aragon, Spain. Implications for vaccination programmes. PLoS ONE.

[40] O’Driscoll M, Ribeiro Dos Santos G, Wang L, Cummings DA, Azman AS, Paireau J, Fontanet A, Cauchemez S, Salje H (2021). Age-specific mortality and immunity patterns of SARS-CoV-2. Nature.

[41] Mallapaty S (2020). The coronavirus is most deadly if you are old and male. Nature.

[42] Dudel C, Riffe T, Acosta E, van Raalte A, Strozza C, Myrskylä M (2020). Monitoring trends and differences in COVID-19 case-fatality rates using decomposition methods: contributions of age structure and age-specific fatality. PLoS ONE.

[43] Hoffmann C, Wolf E (2021). Older age groups and country-specific case fatality rates of COVID-19 in Europe, USA and Canada. Infection.

[44] Dowd JB, Andriano L, Brazel DM, Rotondi V, Block P, Ding X, Liu Y, Mills MC (2020). Demographic science aids in understanding the spread and fatality rates of COVID-19. Proceedings of the National Academy of Sciences..

[45] Dalal J, Triulzi I, James A, Nguimbis B, Dri GG, Venkatasubramanian A, Royd LN, Mesa SB, Somerville C, Turchetti G, Stoll B (2021). COVID-19 mortality in women and men in sub-Saharan Africa: a cross-sectional study. BMJ global health..

[46] Haider N, Yavlinsky A, Chang YM, Hasan MN, Benfield C, Osman AY, Uddin MJ, Dar O, Ntoumi F, Zumla A (2020). The Global Health Security index and joint external evaluation score for health preparedness are not correlated with countries’ COVID-19 detection response time and mortality outcome. Epidemiol Infect.

[47] Stokes EK, Zambrano LD, Anderson KN, Marder EP, Raz KM, Felix SEB, Tie Y, Fullerton KE (2020). Coronavirus disease 2019 case surveillance—United States, january 22–may 30, 2020. Morb Mortal Wkly Rep.

[48] Mohammad S, Aziz R, Al Mahri S, Malik SS, Haji E, Khan AH, Khatlani TS, Bouchama A (2021). Obesity and COVID-19: what makes obese host so vulnerable?. Immun Ageing.

[49] Kousi T, Vivacqua D, Dalal J, James A, Câmara DC, Mesa SB, Chimbetete C, Impouma B, Williams GS, Mboussou F, Mlanda T (2022). COVID-19 pandemic in Africa’s island nations during the first 9 months: a descriptive study of variation in patterns of infection, severe disease, and response measures. BMJ global health..

[50] Ren R, Zhang Y, Li Q, McGoogan JM, Feng Z, Gao GF, Wu Z (2021). Asymptomatic SARS-CoV-2 infections among persons entering China from April 16 to October 12, 2020. JAMA.

[51] Kenyon C (2020). Flattening-the-curve associated with reduced COVID-19 case fatality rates- an ecological analysis of 65 countries. J Infect.

[52] Muniyappa R, Wilkins KJ (2020). Diabetes, obesity, and risk prediction of severe COVID-19. J Clin Endocrinol Metabolism.

[53] Syed AA, Soran H, Adam S. Obesity and covid-19: the unseen risks. BMJ. 2020;370:m2823. 10.1136/bmj.m2823 32675216

[54] Brooke J, Jackson D. Older people and COVID-19 isolation, risk and ageism. J Clin Nurs 2020. 10.1111/jocn.15274 32239784

[55] Foo O, Hiu S, Teare D, Syed AA, Razvi S (2021). A global country-level analysis of the relationship between obesity and COVID-19 cases and mortality. Diabetes Obes Metab.

[56] Wise J (2021). Covid-19: highest death rates seen in countries with most overweight populations. BMJ.

[57] Oshakbayev K, Zhankalova Z, Gazaliyeva M, Mustafin K, Bedelbayeva G, Dukenbayeva B, Otarbayev N, Tordai A (2022). Association between COVID-19 morbidity, mortality, and gross domestic product, overweight/ obesity, non-communicable diseases, vaccination rate: a cross-sectional study. J Infect Public Health.

[58] Price-Haywood EG, Burton J, Fort D, Seoane L (2020). Hospitalization and mortality among black patients and white patients with Covid-19. N Engl J Med.

[59] Poly TN, Islam MM, Yang HC, Lin MC, Jian WS, Hsu MH, Jack Li YC (2021). Obesity and mortality among patients diagnosed with COVID-19: a systematic review and meta-analysis. Frontiers in medicine..

[60] Hamer M, Gale CR, Kivimäki M, Batty GD (2020). Overweight, obesity, and risk of hospitalization for COVID-19: A community-based cohort study of adults in the United Kingdom. Proceedings of the National Academy of Sciences.

[61] Steele RM, Finucane FM, Griffin SJ, Wareham NJ, Ekelund U (2009). Obesity is associated with altered lung function independently of physical activity and fitness. Obes (Silver Spring Md).

[62] Lee H, Lee JR, Jung H, Lee JY (2021). Power of universal health coverage in the era of COVID-19: a nationwide observational study. Lancet Reg health Western Pac.

[63] Dongarwar D, Salihu HM (2020). COVID-19 pandemic: marked global disparities in fatalities according to geographic location and universal health care. Int J Maternal Child Health AIDS.

[64] Varkey RS, Joy J, Sarmah G, Panda PK (2020). Socioeconomic determinants of COVID-19 in Asian countries: An empirical analysis. J Public Affairs..

[65] Mogi R, Spijker J (2022). The influence of social and economic ties to the spread of COVID-19 in Europe. J Popul Res..

[66] Martins-Filho PR (2021). Relationship between population density and COVID-19 incidence and mortality estimates: a county-level analysis. J Infect Public Health.

[67] Kodera S, Rashed EA, Hirata A (2020). Correlation between COVID-19 morbidity and mortality rates in Japan and local population density, temperature, and absolute humidity. Int J Environ Res Public Health.

[68] Bhadra A, Mukherjee A, Sarkar K (2021). Impact of population density on Covid-19 infected and mortality rate in India. Model Earth Syst Environ.

[69] Sy KTL, White LF, Nichols BE (2021). Population density and basic reproductive number of COVID-19 across United States counties. PLoS ONE.

[70] Van Dorn A, Cooney RE, Sabin ML (2020). COVID-19 exacerbating inequalities in the US. Lancet (London England).

[71] Ji Y, Ma Z, Peppelenbosch MP, Pan Q (2020). Potential association between COVID-19 mortality and health-care resource availability. The Lancet Global health.

[72] King JS (2020). Covid-19 and the need for health care reform. N Engl J Med.

[73] Sigler T, Mahmuda S, Kimpton A, Loginova J, Wohland P, Charles-Edwards E, Corcoran J (2021). The socio-spatial determinants of COVID-19 diffusion: the impact of globalisation, settlement characteristics and population. Globalization and health.

[74] James A, Dalal J, Kousi T, Vivacqua D, Câmara DC, Dos Reis IC, Mesa SB, Ng’ambi W, Ansobi P, Bianchi LM, Lee TM. An in-depth statistical analysis of the COVID-19 pandemic’s initial spread in the WHO African region. BMJ global health. 2022;7(4):e007295. 10.1136/bmjgh-2021-007295 PMC9013786 35418411

[75] Lee Y, Kim S, Oh J, Lee S (2022). An ecological study on the association between International Health Regulations (IHR) core capacity scores and the Universal Health Coverage (UHC) service coverage index. Globalization and health.

[76] Assefa Y, Hill PS, Gilks CF, Damme WV, Pas Rvd, Woldeyohannes S, Reid S (2020). Global health security and universal health coverage: understanding convergences and divergences for a synergistic response. PLoS ONE.

[77] Immovilli P, Morelli N, Rota E, Guidetti D (2021). COVID-19 mortality and health-care resources: Organization. Med intensiva.

[78] World Health Organization. Coronavirus disease (COVID-19) Situation Report, 182. 2020.

[79] Luo G, Zhang X, Zheng H, He D (2021). Infection fatality ratio and case fatality ratio of COVID-19. Int J Infect diseases: IJID : official publication Int Soc Infect Dis.

