# Supplementary material for: The role of universal health coverage and global health security nexus and interplay on SARS-CoV-2 infection and case-fatality rates in Africa : a structural equation modeling approach
Source: Global Health. 2023 Jul 6;19:46. doi: 10.1186/s12992-023-00949-2 (PMC10327394; doi:10.1186/s12992-023-00949-2)
Supplement: Supplementary file 1 — Additional file 1: Appendix 1. List of Africa countries included in this study [file 12992_2023_949_MOESM1_ESM.doc]

**Appendix 1: List of Africa countries included in this study**

| **Country** | **Sub region** | **By income** |
| --- | --- | --- |
| Algeria | Northern Africa | Lower middle income |
| Angola | Middle Africa | Lower middle income |
| Benin | Western Africa | Lower middle income |
| Botswana | Southern Africa | Upper middle income |
| Burkina Faso | Western Africa | Low income |
| Burundi | Eastern Africa | Low income |
| Cabo Verde | Western Africa | Lower middle income |
| Cameroon | Middle Africa | Lower middle income |
| Central African Republic | Middle Africa | Low income |
| Chad | Middle Africa | Low income |
| Comoros | Eastern Africa | Lower middle income |
| Congo, Dem. Rep | Middle Africa | Low income |
| Congo | Middle Africa | Lower middle income |
| Côte d'Ivoire | Western Africa | Lower middle income |
| Djibouti | Eastern Africa | Lower middle income |
| Egypt | Northern Africa | Lower middle income |
| Equatorial Guinea | Middle Africa | Upper middle income |
| Eritrea | Eastern Africa | Low income |
| Eswatini | Southern Africa | Lower middle income |
| Ethiopia | Eastern Africa | Low income |
| Gabon | Middle Africa | Upper middle income |
| Gambia | Western Africa | Low income |
| Ghana | Western Africa | Lower middle income |
| Guinea | Western Africa | Low income |
| Guinea-Bissau | Western Africa | Low income |
| Kenya | Eastern Africa | Lower middle income |
| Liberia | Western Africa | Low income |
| Libya | Northern Africa | Upper middle income |
| Lesotho | Southern Africa | Lower middle income |
| Madagascar | Eastern Africa | Low income |
| Malawi | Eastern Africa | Low income |
| Mali | Western Africa | Low income |
| Mauritania | Western Africa | Lower middle income |
| Mauritius | Eastern Africa | Upper middle income |
| Morocco | Northern Africa | Lower middle income |
| Mozambique | Eastern Africa | Low income |
| Namibia | Southern Africa | Upper middle income |
| Niger | Western Africa | Low income |
| Nigeria | Western Africa | Lower middle income |
| Rwanda | Eastern Africa | Low income |
| Sao Tome and Principe | Middle Africa | Lower middle income |
| Senegal | Western Africa | Lower middle income |
| Seychelles | Eastern Africa | High-income |
| Sierra Leone | Western Africa | Low income |
| Somalia | Eastern Africa | Low income |
| South Africa | Southern Africa | Upper middle income |
| South Sudan | Eastern Africa | Low income |
| Sudan | Northern Africa | Low income |
| Tanzania | Eastern Africa | Lower middle income |
| Togo | Western Africa | Low income |
| Tunisia | Northern Africa | Lower middle income |
| Uganda | Eastern Africa | Low income |
| Zambia | Eastern Africa | Lower middle income |
| Zimbabwe | Eastern Africa | Lower middle income |
