# Supplementary material for: The role of universal health coverage and global health security nexus and interplay on SARS-CoV-2 infection and case-fatality rates in Africa : a structural equation modeling approach
Source: Global Health. 2023 Jul 6;19:46. doi: 10.1186/s12992-023-00949-2 (PMC10327394; doi:10.1186/s12992-023-00949-2)
Supplement: Supplementary file 2 — Additional file 2: Appendix 2. Explanatory variables, descriptions and sources [file 12992_2023_949_MOESM2_ESM.doc]

**Appendix 2**: Explanatory variables, descriptions and sources

| Explanatory variables | Definitions | Source of data |
| --- | --- | --- |
| Universal health coverage (UHC) Service coverage indicators (SCI) components: Service capacity and access | Coverage of essential health services (defined as the average coverage of essential services based on tracer interventions that include basic hospital access; health worker density; access to essential medicines and compliance with the international health regulations, among the general and the most disadvantaged population). | WHO Global Health Observatory indicator |
| UHC SCI components: Noncommunicable diseases | Coverage of essential health services (defined as the average coverage of essential services based on tracer interventions that Includes prevalence of raised blood pressure; mean fasting plasma glucose; cervical cancer screening; tobacco control and best-practice policy implemented for industrially produced trans-fatty acids (TFA), among the general and the most disadvantaged population). | WHO Global Health Observatory indicator |
| UHC SCI components: Reproductive, maternal, newborn and child health | Coverage of essential health services (defined as the average coverage of essential services based on tracer interventions that covers family planning; antenatal care; full child immunization and health-seeking behavior for child illness  , among the general and the most disadvantaged population). | WHO Global Health Observatory indicator |
| UHC SCI components: Infectious diseases | Coverage of essential health services (defined as the average coverage of essential services based on tracer interventions that comprise of tuberculosis effective treatment; HIV antiretroviral treatment; insecticide-treated nets coverage for malaria prevention and adequate sanitation, among the general and the most disadvantaged population). | WHO Global Health Observatory indicator |
| Prevent/Prevention | Prevention of the emergence or release of pathogens | https://www.ghsindex.org/about/ |
| Detect/ Detection and Reporting: | Early detection and reporting for epidemics of potential international concern | https://www.ghsindex.org/about/ |
| Respond/ Rapid Response | Rapid response to and mitigation of the spread of an epidemic | https://www.ghsindex.org/about/ |
| Health/ Health System: | Sufficient and robust health system to treat the sick and protect health workers | https://www.ghsindex.org/about/ |
| Norm/Compliance with International Norms | Commitments to improving national capacity, financing plans to address gaps, and adhering to global norms | https://www.ghsindex.org/about/ |
| Risk/Risk Environment | Overall risk environment and country vulnerability to biological threats | https://www.ghsindex.org/about/ |
| Ambient and household air pollution attributable death rate (per 100,000 population) | The mortality attributable to the joint effects of household and ambient air pollution can be expressed as : Number of deaths Death rate Death rates are calculated by dividing the number of deaths by the total population (or indicated if a different population group is used, e.g. children under 5 years). Evidence from epidemiological studies have shown that exposure to air pollution is linked, among others, to the important diseases considered in this estimate: Acute respiratory infections (estimated for all ages ); Cerebrovascular diseases in adults (estimated above 25 years); Ischaemic heart diseases in adults (estimated above 25 years); Chronic obstructive pulmonary disease in adults (estimated above 25 years); and Lung cancer in adults (estimated above 25 years). | WHO Global Health Observatory indicator |
| Current health expenditure (CHE) as percentage of gross domestic product (GDP) (%) | Health spending measures the final consumption of health care goods and services (i.e. current health expenditure) including personal health care (curative care, rehabilitative care, long-term care, ancillary services and medical goods) and collective services (prevention and public health services as well as health administration), but excluding spending on investments. Health care is financed through a mix of financing arrangements including government spending and compulsory health insurance. | World Health Organization Global Health Expenditure database |
| GDP per capita (current US$)  . | Gross domestic product (GDP) is the standard measure of the value added created through the production of goods and services in a country during a certain period. As such, it also measures the income earned from that production, or the total amount spent on final goods and services (less imports). | World Bank national accounts data, and OECD National Accounts data files |
| Prevalence of obesity among adults | Percentage of defined population with a body mass index (BMI) of 30 kg/m2 or higher among adults | WHO Global Health Observatory indicator |
